# Severe Iatrogenic Tricuspid Regurgitation

**DOI:** 10.7759/cureus.108827

**Published:** 2026-05-14

**Authors:** Inês Amaral Pinto, Diogo Macedo, Rita Rei Neto

**Affiliations:** 1 Internal Medicine, Unidade Local de Saúde Gaia e Espinho, Vila Nova de Gaia, PRT

**Keywords:** cabergoline-associated valvulopathy, cabergoline treatment, ectopic prolactinoma, iatrogenic complication, prolactinoma, tricuspid valve regurgitation

## Abstract

Tricuspid regurgitation (TR) is a valvular heart disease mostly associated with structural heart disease or pulmonary hypertension (PH). Rare cases of TR secondary to exposure to specific medications, such as dopamine agonists, have been described.

A 50-year-old woman presented with a pituitary microprolactinoma (without surgical indication) and a history of pulmonary embolism (PE) in 2021 (risk factors included bromocriptine, which was switched to cabergoline). An echocardiogram performed one year after the PE showed no significant abnormalities. She was maintained on rivaroxaban 10 mg due to suspected chronic PE on CT angiography. She remained asymptomatic until April 2023, when she developed predominantly right-sided heart failure with dyspnoea, peripheral oedema, and ascites. She was admitted for intravenous diuretic therapy and investigation of PH, with mild symptomatic improvement. Diagnostic work-up revealed right chamber dilatation and severe TR due to annular dilatation with tethering of the subvalvular apparatus and thickened leaflets on transthoracic echocardiography, confirmed by transoesophageal echocardiography and cardiac magnetic resonance imaging. Ventilation-perfusion scintigraphy excluded chronic PE, and right heart catheterisation ruled out PH. After exclusion of alternative causes of severe TR and considering the valvular morphology and exposure to cabergoline, the drug was discontinued. Due to persistent NYHA class II heart failure symptoms, the patient underwent tricuspid valvuloplasty via minithoracotomy and has remained asymptomatic since.

This case describes new-onset severe TR with right-sided heart failure in the absence of PH or chronic PE. Withdrawal of cabergoline and optimisation of volume resulted in modest clinical improvement, followed by surgical intervention with full functional recovery. This case highlights the importance of regular clinical and echocardiographic surveillance in patients receiving long-term dopamine agonist therapy, particularly cabergoline, due to the risk of valvulopathy, enabling early diagnosis and timely therapeutic intervention.

## Introduction

Tricuspid regurgitation (TR) is a relatively common valvular disorder, most often secondary to structural heart disease, pulmonary hypertension (PH), or right ventricular dilatation. However, iatrogenic causes, particularly related to dopamine agonists, represent a rare but clinically relevant entity [1]. The development of this form of valvular heart disease appears to be related to the affinity of these drugs for serotonin 5-HT₂B receptors expressed on cardiac valve leaflets. Chronic activation of these receptors induces fibroblast proliferation and extracellular matrix deposition, leading to leaflet thickening and retraction, with subsequent valvular regurgitation [1-3].

The risk of cabergoline-associated valvulopathy (CAV) was initially described in patients with Parkinson’s disease receiving high cumulative doses. Subsequent observational studies demonstrated a significant association between prolonged exposure to dopamine agonists and valvular regurgitation, particularly affecting the mitral and aortic valves [4-8].

We present a case of severe iatrogenic TR occurring after prolonged treatment with cabergoline at a moderate cumulative dose in the context of a prolactinoma, contributing to the limited existing literature on this rare complication. To date, only a very limited number of reports have described significant involvement of the tricuspid valve with morphological features compatible with CAV [9-11].

## Case presentation

A 50-year-old woman initially presented with headache and secondary amenorrhoea at the age of 25. Serum prolactin levels were 102 ng/mL (reference range: 4.79-23.3 ng/mL), and contrast-enhanced MRI demonstrated a 5.4-mm lesion in the adenohypophysis, consistent with a microprolactinoma. She was initially treated with bromocriptine 5 mg daily and remained on this therapy for 20 years, with stable imaging findings and prolactin levels <20 ng/mL.

In 2021, at the age of 45, she was diagnosed with low-risk pulmonary embolism (PE). Given the reported association of bromocriptine with an increased risk of venous thromboembolism, this medication was discontinued, and cabergoline 0.5 mg once weekly was initiated. Due to persistently elevated prolactin levels, the dose was progressively titrated to 1 mg twice weekly, with the last adjustment in February 2023.

She was followed in the internal medicine clinic for venous thromboembolism, and a transthoracic echocardiogram performed one year after the PE showed no evidence of valvular disease. She remained anticoagulated with rivaroxaban 10 mg daily due to suspected chronic PE on chest CT angiography.

The patient remained asymptomatic until April 2023, when she developed progressive dyspnoea, reaching NYHA class III, along with bilateral symmetrical lower limb oedema extending to the thighs and mild ascites over a three-month period, significantly limiting daily activities. She was admitted in August 2023 for investigation and symptomatic management with intravenous loop diuretics, resulting in symptomatic improvement.

**Figure 1:**
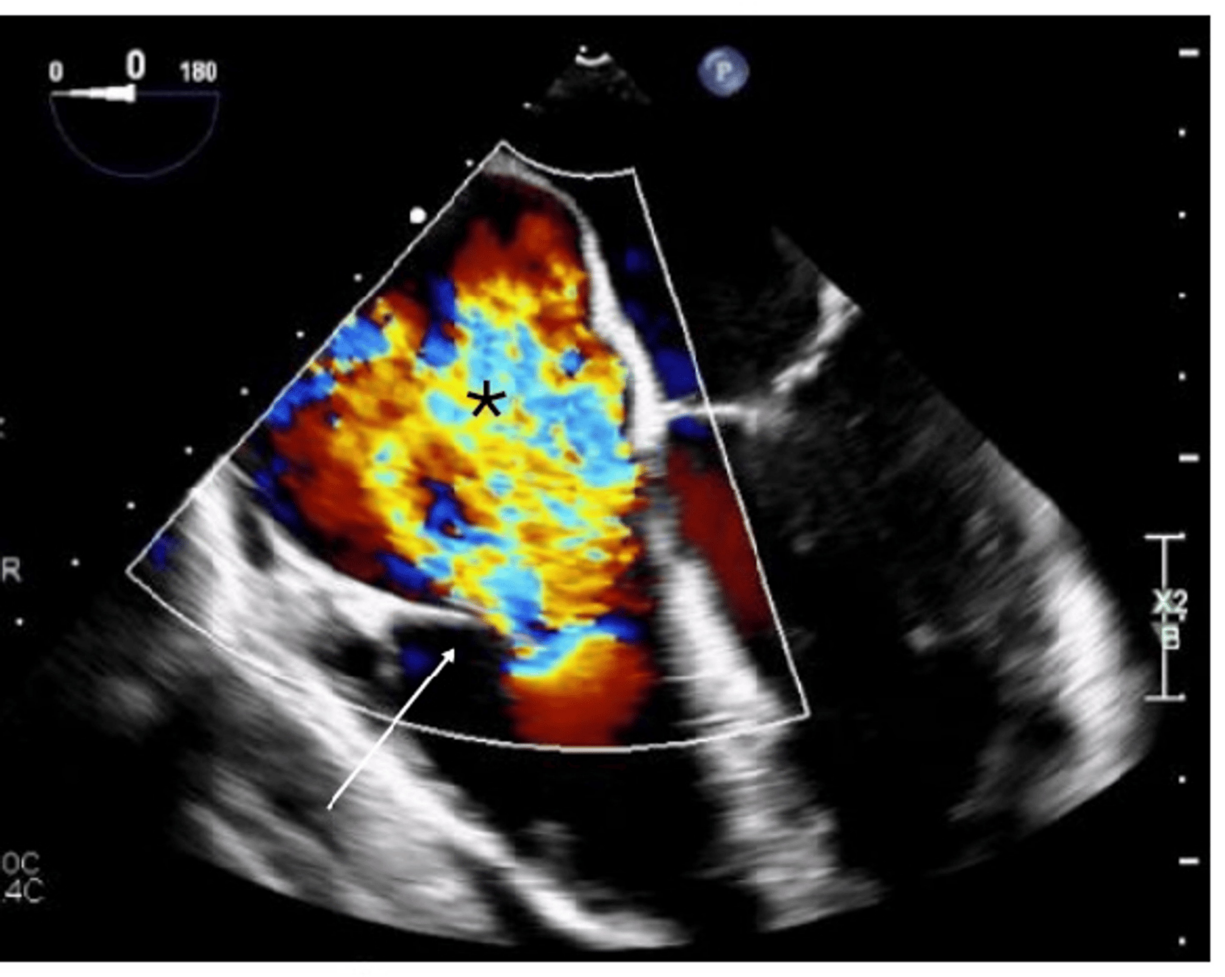
Transoesophageal echocardiography showing mild right atrial dilatation (*), tricuspid annular dilatation with severe tricuspid regurgitation (arrow) (effective regurgitant orifice area of 0.7 cm², regurgitant volume of 57 mL) and preserved biventricular function.

Transoesophageal echocardiography revealed severe right chamber dilatation (indexed volume 50 mL/m²; basal RV diameter 47 mm; mid-cavity diameter 32 mm) and a tricuspid valve with mild annular dilatation and markedly retracted leaflets, resulting in an approximately 8-mm coaptation defect and severe (grade IV) TR (Figure 1). The estimated pulmonary artery systolic pressure was 44 mmHg. Ventilation-perfusion scintigraphy excluded chronic PE.

Right heart catheterisation demonstrated normal pulmonary artery pressures, excluding PH. High-resolution chest CT, pulmonary function tests, and morphological cardiac MRI revealed no relevant abnormalities.

Cabergoline was discontinued and she was referred for cardiothoracic surgery after two months of oral diuretic therapy. In October 2023, she underwent a tricuspid valvuloplasty via minithoracotomy and she remained asymptomatic.

Regarding the microprolactinoma, she continues follow-up in the endocrinology clinic, with stable lesion size and prolactin levels of 70-80 ng/mL, remaining asymptomatic and currently not receiving medical therapy.

## Discussion

This case illustrates new-onset severe TR in the absence of PH, chronic PE, or structural heart disease, developing after approximately two years of progressively increased cabergoline therapy (estimated cumulative dose: 64 mg).

The differential diagnosis of severe TR includes functional causes (annular dilatation due to PH or left-sided heart disease), infectious causes (endocarditis), and degenerative disease. In this case, the absence of PH, chronic PE, and alternative structural abnormalities strongly supports an iatrogenic aetiology. Echocardiography demonstrated thickened and retracted tricuspid leaflets without calcification or masses, consistent with primary valvular involvement and typical of CAV.

Confirmed cases of CAV requiring intervention are rare. Cawood et al. (2009) described one of the first cases in a patient with prolactinoma treated with low-dose cabergoline, involving mitral valve thickening [9]. Caputo et al. (2018) reported the third histologically confirmed case of CAV [10], involving multiple valves after very high cumulative exposure (>4,000 mg). More recently, Hayes et al. (2023) reported the first case of significant tricuspid valvulopathy associated with cabergoline in prolactinoma, characterized by severe regurgitation, leaflet thickening and restriction, and exclusion of alternative causes [11].

The present case shares important clinical similarities with that reported by Hayes et al., representing an additional example of predominant tricuspid valve involvement at a moderate cumulative dose, reinforcing that the risk of CAV is not restricted to high-dose exposure. Functional recovery after drug withdrawal and subsequent surgical repair supports the potential reversibility of this condition [2,3].

Compared with cabergoline, bromocriptine has a markedly lower affinity for 5-HT₂B receptors and has not been consistently associated with clinically significant valvular fibrosis, which may explain the absence of valvular disease during the patient’s long-term exposure to bromocriptine prior to cabergoline initiation [5].

Given the limited number of documented cases, periodic echocardiographic surveillance is recommended in patients receiving long-term cabergoline therapy. Current guidelines suggest transthoracic echocardiography every 12-24 months, with frequency adjusted according to dose and treatment duration [12].

## Conclusions

This case reinforces the need for a high level of suspicion for iatrogenic valvulopathy in patients treated with cabergoline, even at therapeutic doses, particularly when symptoms of heart failure develop. Early recognition allows treatment modification and may prevent irreversible complications.
